# Intrauterine Inflammation and Maternal Exposure to Ambient PM_2.5_ during Preconception and Specific Periods of Pregnancy: The Boston Birth Cohort

**DOI:** 10.1289/EHP243

**Published:** 2016-04-27

**Authors:** Rebecca Massa Nachman, Guangyun Mao, Xingyou Zhang, Xiumei Hong, Zhu Chen, Claire Sampankanpanich Soria, Huan He, Guoying Wang, Deanna Caruso, Colleen Pearson, Shyam Biswal, Barry Zuckerman, Marsha Wills-Karp, Xiaobin Wang

**Affiliations:** 1 Department of Environmental Health Sciences, Johns Hopkins Bloomberg School of Public Health, Baltimore, Maryland, USA; 2 School of Environmental Science & Public Health, Wenzhou Medical University, Wenzhou, China; 3 Center on Clinical and Epidemiological Eye Research, the Affiliated Eye Hospital of Wenzhou Medical University, Wenzhou, China; 4 Department of Population, Family and Reproductive Health, Center on the Early Life Origins of Disease, Johns Hopkins Bloomberg School of Public Health, Baltimore, USA; 5 Mary Ann and J. Milburn Smith Child Health Research Program, Children’s Memorial Research Center, Chicago, Illinois, USA; 6 Department of Pediatrics, Boston University School of Medicine and Boston Medical Center, Boston, USA

## Abstract

**Background:**

Prenatal exposure to ambient PM2.5, (i.e., fine particulate matter, aerodynamic diameter ≤ 2.5 μm) has been associated with preterm birth and low birth weight. The association between prenatal PM2.5 exposure and intrauterine inflammation (IUI), an important risk factor for preterm birth and neurodevelopmental outcomes, has not been evaluated.

**Objectives:**

We aimed to investigate the association between maternal exposure to PM2.5 and IUI in the Boston Birth Cohort, a predominantly urban low-income minority population.

**Methods:**

This analysis included 5,059 mother–infant pairs in the Boston Birth Cohort. IUI was assessed based on intrapartum fever and placenta pathology. PM2.5 exposure was assigned using data from the U.S. EPA’s Air Quality System. Odds ratios (OR) and 95% confidence intervals (CI) quantified the association of maternal PM2.5 exposure during preconception and various periods of pregnancy with IUI.

**Results:**

Comparing the highest with the lowest PM2.5 exposure quartiles, the multi-adjusted association with IUI was significant for all exposure periods considered, including 3 months before conception (OR = 1.52; 95% CI: 1.22, 1.89), first trimester (OR = 1.93; 95% CI: 1.55, 2.40), second trimester (OR = 1.67; 95% CI: 1.35, 2.08), third trimester (OR = 1.53; 95% CI: 1.24, 1.90), and whole pregnancy (OR = 1.92; 95% CI: 1.55, 2.37).

**Conclusions:**

Despite relatively low exposures, our results suggest a monotonic positive relationship between PM2.5 exposure during preconception and pregnancy and IUI. IUI may be a sensitive biomarker for assessing early biological effect of PM2.5 exposure on the developing fetus.

**Citation:**

Nachman RM, Mao G, Zhang X, Hong X, Chen Z, Soria CS, He H, Wang G, Caruso D, Pearson C, Biswal S, Zuckerman B, Wills-Karp M, Wang X. 2016. Intrauterine inflammation and maternal exposure to ambient PM2.5 during preconception and specific periods of pregnancy: the Boston Birth Cohort. Environ Health Perspect 124:1608–1615; http://dx.doi.org/10.1289/EHP243

## Introduction

Maternal exposure to air pollution during pregnancy is associated with adverse birth outcomes such as low birth weight and preterm birth (Bell et al. 2007; Brauer et al. 2008; Dadvand et al. 2014; Fleischer et al. 2014; Gehring et al. 2011; Jalaludin et al. 2007; Kloog et al. 2012; Le et al. 2012; Lee et al. 2013; Malmqvist et al. 2011; Pereira et al. 2014; Ritz et al. 2000, 2007; Wang et al. 1997; Xu et al. 1995). The biological mechanisms behind this relationship are not well understood, but inflammation is thought to play a role (Muglia and Katz 2010; Slama et al. 2008). Exposure to PM_2.5_ (particulate matter with an aerodynamic diameter ≤ 2.5 μm) and resulting oxidative stress may lead to chronic systematic inflammation (Hajat et al. 2015; WHO 2003). Maternal PM_2.5_ exposure and inflammation during pregnancy (Lee et al. 2011; van den Hooven 2012a), may affect the growth, development, and function of the placenta (Backes et al. 2013; van den Hooven 2012b; Wright and Brunst 2013). Emerging evidence in rats suggests that PM_2.5_ exposure of the pregnant mother may induce inflammation at the site of the placenta (de Melo et al. 2015), raising concerns that PM_2.5_ may be associated with intrauterine inflammation (IUI), a known risk factor for preterm birth, low birth weight, and poor respiratory outcomes in early childhood [Gupta et al. 2007; Institute of Medicine (U.S.) Committee on Understanding Premature Birth and Assuring Healthy Outcomes 2007; Kumar et al. 2008; Mestan et al. 2010]. In humans, cord blood C-reactive protein concentrations—evidence of systemic inflammation in the fetus—have been positively associated with maternal exposure to particulate matter during pregnancy, and IUI is hypothesized to play a role (van den Hooven et al. 2012a). However, currently, to our knowledge, no investigation of the association between air pollution exposure and IUI has been carried out. Large cohorts created through the linkage of birth registries with air pollution data are useful for the study of preterm birth and low birth weight, because these outcomes can be identified using data commonly included in birth records. However, study of IUI is complicated by the need for tissue samples and/or clinical data from which the presence of IUI can be determined.

In addition, few existing studies have investigated the reproductive effects of air pollution in one of the most at-risk populations, urban minorities (Le et al. 2012). Within the United States, African Americans and Hispanics are more highly exposed to air pollution (Jones et al. 2014), and African Americans have higher rates of IUI than do whites [Institute of Medicine (U.S.) Committee on Understanding Premature Birth and Assuring Healthy Outcomes 2007]. Estimates of the prevalence of IUI range from 25% to 50% of preterm births (Culhane and Goldenberg 2011; Goldenberg et al. 2000, 2008; Incerpi 2010), and 20% of full-term births in the general population (Incerpi 2010), though data are sparse because evaluation of IUI by adequately sensitive methods is not routinely performed.

In this study, we assessed the relationship between IUI and maternal exposure to ambient PM_2.5_ before and during pregnancy in an at-risk urban predominantly minority population. By examining IUI, we potentially bridge two previously studied relationships: PM_2.5_ exposure and preterm birth (Rappazzo et al. 2014; Ritz et al. 2007; Pereira et al. 2014; Xu et al. 1995), and IUI and preterm birth (Goldenberg et al. 2000). The study of IUI will further our understanding of inflammation as a potential marker of biologic effect of exposure to PM_2.5_ early in life with relevance to the health of the developing fetus.

## Methods

### Study Design and Population

The study population was a subgroup of mother–infant pairs recruited from 1999 through 2012 as part of the Boston Birth Cohort, an ongoing prospective cohort established in 1998 at the Boston Medical Center (BMC). BMC serves an ethnically diverse community of patients who primarily reside in an urban setting, and the birth cohort is enriched for preterm birth by recruiting at a ratio of approximately one preterm for two full-term births. Multiple births and newborns with major birth defects were excluded. Patient recruitment and data collection methods are detailed elsewhere (Wang et al. 2002). Briefly, recruitment took place 24–48 hr after birth, and informed written consent was obtained from all participating mothers. Clinical data were obtained from maternal and infant medical records, and an interview questionnaire was administered at the time of recruitment and consent to determine social demographic variables, smoking status, and alcohol intake. In addition, maternal blood, placental tissue, and other biological samples were collected at the time of recruitment and analyzed or stored for later analysis. The study protocol was approved by institutional review boards at the BMC and Johns Hopkins Bloomberg School of Public Health.

### Data Collection

Detailed data collection and measurement methods for clinical and sociodemographic variables have been previously published elsewhere (Kumar et al. 2008; Wang et al. 2002). Briefly, an interview was conducted using a standardized questionnaire, upon obtaining informed signed consent from the mother, from which data on maternal education, household income, current and previous residential addresses, and maternal smoking before and during the pregnancy were obtained for the period 3 months before conception and for each of the first, second, and third trimesters. Participants were asked to provide dates of residence changes. In addition, a standardized abstraction form was used to obtain data such as maternal prepregnancy body mass index (BMI), maternal age at delivery, sex of the baby, ultrasound findings, pathology reports, laboratory results, and labor and delivery course from medical records. Medical records were also used to identify changes in residential address not captured in the questionnaire. Gestational age was assessed based on the date of the last menstrual period as well as results of early ultrasound (< 20 weeks gestation), abstracted from medical records.

### Outcome Assessment

Mothers were defined as having IUI if either of the following criteria were met: placenta histopathology consistent with uterine inflammation, or the presence of intrapartum maternal fever > 38°C at parturition. Placentas were obtained by the labor and delivery nurses at the time of delivery and sent to the hospital perinatal pathologist to be processed and reviewed. The presence of inflammation (acute or chronic) in any of several locations in the placenta, including the decidua, chorion, amnion, chorionic plate, and the umbilical cord, was reported according to algorithms consistent with guidelines of the College of American Pathologists (Benirschke et al. 2000; Langston et al. 1997). Strong intra-observer agreement (κ = 0.78–0.81) has been reported for the diagnosis of inflammatory conditions such as chorioamnionitis (Simmonds et al. 2004). During the course of the Boston Birth Cohort, a new hospital pathologist took over examination of placentas. Before this, for training purposes, a subset (*n* = 298) of the placental pathology slides was randomly selected and independently reviewed by the two placental pathologists, who compared readings and reached consensus about the reporting of the pathology findings.

### Exposure Assessment

**Figure 1:**
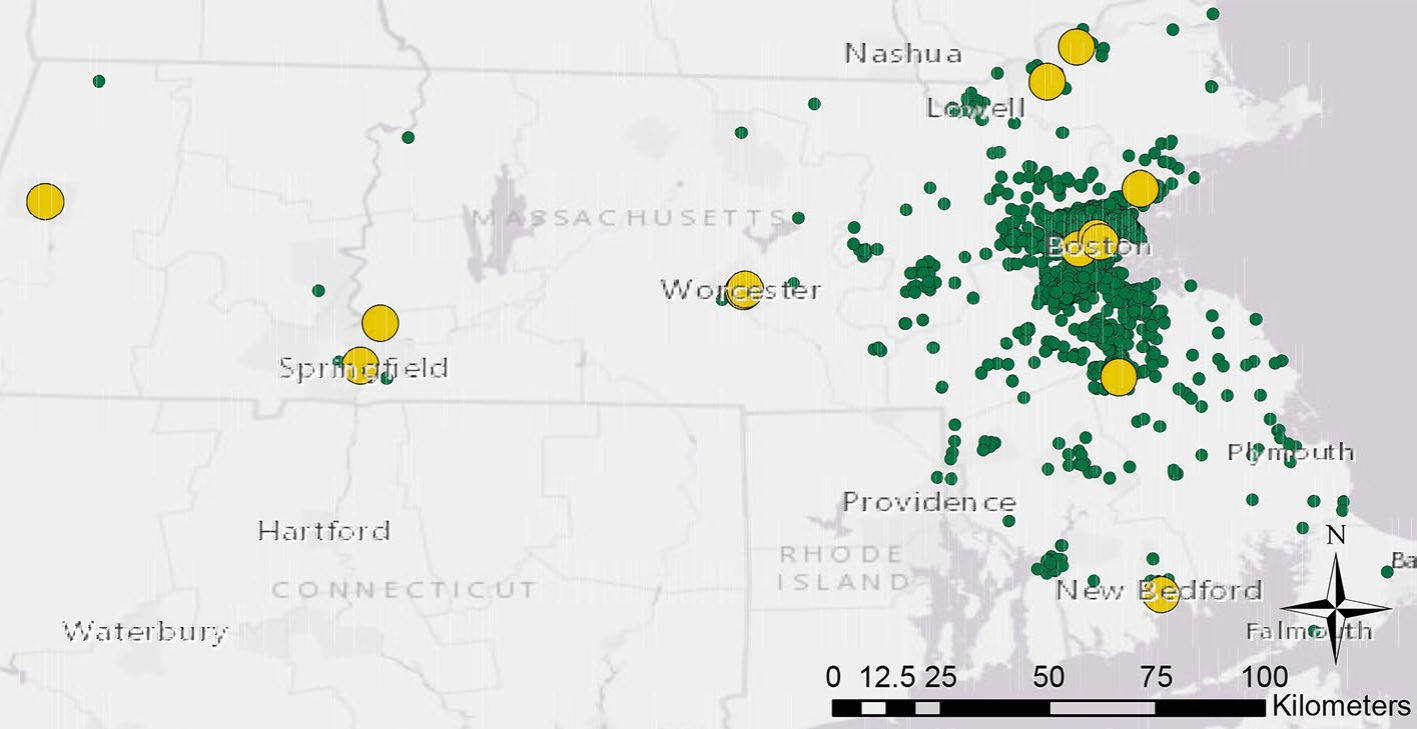
Map of the study area (Boston, Massachusetts) depicting distances between 5,059 mother–infant pairs (green) and PM_2.5_ air quality monitors (yellow) included in the study. (Map image is the intellectual property of ESRI and is used herein under license. Copyright © 2014 ESRI and its licensors. All rights reserved.)

Individual exposures to PM_2.5_ were estimated by assigning each subject to the closest monitor by their residential address, reported at the time of study recruitment, using ArcGIS 10.2 (ESRI, Inc.). No limits were placed on the distance between subjects and monitors. A map of the study area depicts the locations of subjects relative to monitor locations (Figure 1). We included only data from monitors that had at least one measurement per week for 75% of the study period. All other monitors were excluded from the analysis. Measurements were recorded every 3 days for the monitors in the study except for a few short periods during which measurements were recorded daily.

Exposure periods were calculated based on the gestational age of the infant at birth and were divided into phases: 3 months (90 days) before pregnancy, first trimester (day 1 to day 90 of pregnancy), second trimester (day 91 to day 180 of pregnancy), third trimester (day 181 of pregnancy to birth), and the last month before delivery. Because data sets from monitors in the study far exceeded the criterion of 1 measurement per week for 75% of the study period, the last recorded measurement was assigned to the next 2 days for those monitors with a 1-in-3 day measurement schedule. Exposure was assessed for each individual participant as the geometric mean of the daily PM_2.5_ ambient concentrations during a given exposure period of interest (e.g., preconception, first, second, and third trimester, whole pregnancy, and last month). Daily PM_2.5_ concentration data came from the monitor closest to the participant’s date-specific address. If a participant moved residence, daily data from the monitor closest to the previous residence were used up to the date of the move, and data from the monitor closest to the new address were used starting on the date of the move. Quartiles of exposure were determined separately for each pregnancy period from the distribution of all participant exposures during that period. Exposure was categorized by quartile and as a continuous variable. Interquartile ranges (IQRs) and cut points between quartiles varied slightly by period of exposure due to missing exposure data, and varied between the main analysis and analyses performed in subsets of the study population.

### Statistical Analysis

Population characteristics among those positive for IUI and negative for IUI were compared as follows. Continuous variables were expressed as median (IQR), and differences between the two groups were assessed by Mann–Whitney *U*-test because variable distributions were skewed. Categorical data were expressed as *n* (%), and a chi-square test was used to compare the differences of the proportion between the two groups.

We examined the associations [odds ratios (OR) and 95% confidence intervals (CI)] of IUI (binary) by quartile of maternal exposure to residential ambient PM_2.5_ using multivariable logistic regression via PROC GLM in SAS 9.3 (SAS Institute Inc.). ORs were also estimated for PM_2.5_ exposure as a continuous variable. The following covariates included in the models were chosen *a priori* based on potential association with PM_2.5_ exposure and because they are known risk factors for either IUI or preterm birth: maternal smoking status, race, BMI, age at delivery, education level, parity, season of delivery, household income, and sex of the baby [Institute of Medicine (U.S.) Committee on Understanding Premature Birth and Assuring Healthy Outcomes 2007; Astolfi et al. 1999]. All covariates were treated as categorical variables; missing data for each covariate were treated as a separate category. Smoking status was self-reported in response to the interview questionnaire administered at enrollment for four time periods: 3 months before conception and first, second, and third trimesters. Based on these data, smoking was grouped into three categories: never smoking, if no smoking was reported during any period of the pregnancy or the 3 months prior; quit smoking if smoking occurred during the first trimester or the 3 months prior, and continued smoking if smoking occurred continued past the first trimester. Also self-reported at enrollment were race/ethnicity, categorized as Hispanic, white, African/African American, or other; prepregnancy BMI, calculated as reported weight divided by height squared (kg/m^2^), and categorized as underweight (BMI < 18.5), normal (18.5 ≤ BMI < 25), overweight (25 ≤ BMI < 30), and obese (BMI ≥ 30); age at delivery, categorized into five levels (< 20, 20–24, 25–29, 30–34, and ≥ 35 years); education, categorized as middle school or below, high school, and college or above; parity, categorized as 0 or ≥ 1 live birth before index birth, and annual income treated as a dichotomous variable with income < $25,000 as the reference group. We applied separate models to each of several exposure timing scenarios: maternal exposure 3 months before pregnancy, during the first trimester, during the second trimester, during the third trimester, during the entire pregnancy, and 1 month before birth. Two-sided *p*-value < 0.05 was the designated significance level. We also examined significance after correcting for multiple comparisons by establishing a more stringent significance level equal to 0.05 divided by 6, the number of exposure periods for which we tested the association between maternal PM_2.5_ and IUI. When taking into account multiple comparisons, effect estimates were considered to be significant if the *p*-value was < 0.008.

In addition, we performed several subanalyses to evaluate the robustness of the findings, the results of which are presented in the Supplemental Material. To account for possible confounding by exposure to PM_2.5_ during other pregnancy periods, we estimated the effect of exposure during each of four exposure periods (preconception, first trimester, second trimester, and third trimester), adjusting for exposure during the other three time periods. If exposure during any of the other three time periods was missing, as it was in subjects who were enrolled near the start or end dates of the study, exposure for the missing periods was assigned to a separate category such that the sample size for each exposure period of interest remained the same in the multiple time–period model as it was in the single time–period model. In addition, we performed a subgroup analysis among participants living within either 4 km or 10 km of a monitor to assess potential bias due to exposure misclassification among participants living greater than those distances from a monitor. We also performed a stratified analysis to examine the consistency or difference of the effect in participants who were African American compared with those who were not. In this analysis, non–African-American participants were grouped to achieve a large enough sample size in both strata. To evaluate modification of the effect by warm or cool season, we performed an additional analysis stratifying subjects exposed from May through September (warm season) and October through April (cool season).

## Results

### Population Characteristics

Of those with IUI (*n* = 910), 229 were assessed based on fever only [i.e., histology was negative (*n* = 82) or missing (*n* = 147)], 563 based on placenta histology only [i.e., fever was negative (*n* = 478) or missing (*n* = 85)], and 118 on both (i.e., histology and fever were positive). Of those with no IUI (*n* = 4,149), 1,412 were negative for both fever and histology, 2,513 were negative for fever and histology was missing, and 224 were negative for histology and fever was missing. Participants for whom both histology and fever data were missing were excluded from the analysis (*n* = 583). Placenta pathology data were available for 47.4% of the study population, and fever data were available for 93.9% of the population. In cases when both variables were available, concordance for presence of IUI was 73.2%. IUI was present in 18.0% of the study population. Of 1,567 mothers delivering preterm, 378 (24.1%) were positive for IUI, compared with 532 (15.2%) of 3,492 mothers who carried to term. Among 579 very preterm (< 34 weeks gestation), 217 (37.48%) were positive for IUI.

Population characteristics by IUI status are presented in Table 1. Mothers with IUI at the time of the birth (compared to those without inflammation) tended to be younger, more likely to be smokers, born in the United States, and nulliparous; they were less likely to be Hispanic or have received a college education or higher (Table 1). The ratio of boys to girls was higher among babies born to mothers with IUI than among those born to mothers with no IUI. Also, gestational age at birth and birth weight were lower among mothers with IUI. There was no significant difference in IUI by income categorized as ≥ $25,000 or < $25,000. Data on household income were missing for 40.0% of participants (40.1% missing among those with IUI and 39.5% among those without IUI).

**Table 1:** Birth-related covariates and characteristics of the study population.

Characteristics	No inflammation (*n* = 4,149)	Inflammation (*n* = 910)	*p*-Value
Maternal characteristics
Age at delivery (years)			0.031
< 20	453 (10.92)	115 (12.64)
20–24	1,030 (24.83)	256 (28.13)
25–29	1,046 (25.21)	223 (24.51)
30–34	923 (22.25)	193 (21.21)
≥ 35	697 (16.80)	123 (13.52)
Prepregnancy BMI (kg/m^2^)			0.737
< 18.5	191 (4.60)	40 (4.40)
18.5–24.9	1,943 (46.83)	430 (47.25)
25.0–29.9	1,150 (27.72)	242 (26.59)
≥ 30.0	771 (18.58)	171 (18.79)
Missing	94 (2.27)	27 (2.97)
Race/ethnicity			0.050
Hispanic	1,183 (28.51)	215 (23.63)
White	460 (11.09)	109 (11.98)
African/African American	2,014 (48.54)	474 (52.09)
Others	487 (11.74)	110 (12.09)
Missing	5 (0.12)	2 (0.22)
Smoking during pregnancy			0.046
Never smoking before	3,287 (79.22)	684 (75.16)
Quit smoking	289 (6.97)	70 (7.69)
Continue smoking	544 (13.11)	147 (16.15)
Missing	29 (0.70)	9 (0.99)
Education category			0.010
Middle school or below	1,395 (33.62)	302 (33.19)
High school	1,353 (32.61)	309 (33.96)
College or above	1,370 (33.02)	282 (30.99)
Missing	31 (0.75)	17 (1.87)
Household income per year			0.811
< $25,000	1,794 (43.24)	404 (44.40)
≥ $25,000	690 (16.63)	147 (16.15)
Missing	1,665 (40.13)	359 (39.45)
Place of birth			< 0.001
Non-USA	2,587 (62.35)	508 (55.82)
USA	1,544 (37.21)	392 (43.08)
Missing	18 (0.43)	10 (1.10)
Season at delivery			0.479
Spring (March–May)	921 (22.20)	212 (23.30)
Summer (June–August)	1,043 (25.14)	213 (23.41)
Autumn (September–November)	1,091 (26.30)	255 (28.02)
Winter (December–February)	1,094 (26.37)	230 (25.27)
Parity			< 0.001
0	1,662 (40.06)	477 (52.42)
≥ 1	2,483 (59.85)	433 (47.58)
Missing	4 (0.10)	0 (0.00)
Children characteristics
Sex			0.003
Girl	2,126 (51.24)	439 (48.24)
Boy	2,023 (48.76)	469 (51.54)
Missing	0 (0.00)	2 (0.22)
Gestational age (weeks)^*a*^	38.57 (36.57, 40.00)	38.14 (34.29, 40.00)	< 0.001
Birth weight (g)^*a*^	2930.0 (2390.0, 3410.0)	2692.5 (2035.0, 3365.0)	< 0.001
^***a***^Data did not meet normal distribution and are described as median (IQR), and Mann–Whitney *U*-test was applied to compare the difference between two groups.			

Among all participants included in this analysis, mean maternal age was 28.01 ± 6.54. By race, African Americans were the largest subgroup (49.2%), followed by Hispanics (27.6%). Education varied in the study population: Approximately one-third were educated at the college level or above, a third had completed some high school, and a third were not educated beyond middle school.

The participation rate has been > 90% among mothers approached for recruitment into the larger parent study. Characteristics of the 2,777 Boston Birth Cohort participants excluded from the current analysis for lack of data on IUI (*n* = 583) or lack of PM_2.5_ exposure data (*n* = 2,194) are found in Table S1 and Figure S1. Those excluded were less likely to be < 25 years of age, African American, ever/current smokers, or educated at the college level. Participants excluded were also more likely to be obese, and more likely to have a household income of ≥ $25,000.

### Exposure to Ambient PM_2.5_

Figure 1, a map of the study area, shows individuals’ approximate locations in relation to ambient monitors. Address change occurred during pregnancy in 11.9% of the subjects; the rest remained at the same residential address throughout the pregnancy.

Out of 26 monitors, 13 fit the criteria for inclusion in the study. The average distance of a subject from the nearest monitor was 7.058 ± 6.909 km, and 95% of the subjects’ residential addresses were within 23.4 km of a monitor. 11.39% of the variability in exposures was attributable to monitor site (i.e., spatial variation in exposures). Temporal variability among women assigned to the same monitor accounted for 88.61% of the total variability.

Ambient PM_2.5_ exposures over the entire pregnancy ranged from 5.54 to 29.00 μg/m^3^, with an IQR of 3.31 μg/m^3^ (Table 2). Exposures decreased over the study period (see Figure S2). Participants who were enrolled within 1 year of the start or end of the study period were consequently missing exposure data for one or more periods before or during pregnancy; therefore, the number of subjects varies by exposure period.

**Table 2:** Associations between maternal exposure to ambient PM_2.5_ (quartile) and odds of IUI in the study population.

PM_2.5_ (μg/m^3^)	*n*	Cases (%)	Crude		Adjusted	
			OR (95%CI)	*p*-Value	OR (95%CI)	*p*-Value
Preconception
1.2–9.06	1,212	182 (15.00)	1.00 (1.00, 1.00)	Referent	1.00 (1.00, 1.00)	Referent
9.07–10.85	1,212	194 (16.00)	1.08 (0.87, 1.34)	0.501	1.10 (0.88, 1.38)	0.487
10.86–12.73	1,213	210 (17.30)	1.18 (0.95, 1.47)	0.125	1.18 (0.94, 1.48)	0.154
12.73–29.00	1,212	255 (21.00)	1.51 (1.22, 1.86)	< 0.001	1.52 (1.22, 1.89)	< 0.001*
Per IQR = 3.67			1.05 (0.97, 1.13)	0.239	1.05 (0.97, 1.14)	0.230
1st trimester
4.16–8.99	1,239	171 (13.80)	1.00 (1.00, 1.00)	Referent	1.00 (1.00, 1.00)	Referent
9.00–10.95	1,228	180 (14.70)	1.07 (0.86, 1.35)	0.543	1.11 (0.88, 1.40)	0.361
10.96–12.72	1,234	225 (18.20)	1.39 (1.12, 1.73)	0.003	1.41 (1.13, 1.77)	0.003*
12.72–27.72	1,233	288 (23.40)	1.90 (1.55, 2.35)	< 0.001	1.93 (1.55, 2.40)	< 0.001*
Per IQR = 3.73			1.27 (1.17, 1.39)	< 0.001	1.30 (1.19, 1.42)	< 0.001*
2nd trimester
4.44–8.97	1,249	183 (14.70)	1.00 (1.00, 1.00)	Referent	1.00 (1.00, 1.00)	Referent
8.98–10.96	1,250	194 (15.50)	1.07 (0.86, 1.33)	0.544	1.07 (0.85, 1.34)	0.568
10.97–12.58	1,246	238 (19.10)	1.38 (1.11, 1.70)	0.003	1.39 (1.11, 1.74)	0.004*
12.58–52.90	1,251	280 (22.40)	1.68 (1.37, 2.06)	< 0.001	1.67 (1.35, 2.08)	< 0.001*
Per IQR = 3.61			1.30 (1.20, 1.42)	< 0.001	1.31 (1.20, 1.43)	< 0.001*
3rd trimester
3.20–8.85	1,244	192 (15.40)	1.00 (1.00, 1.00)	Referent	1.00 (1.00, 1.00)	Referent
8.86–10.85	1,244	187 (15.00)	0.97 (0.78, 1.21)	0.780	0.99 (0.79, 1.24)	0.902
10.86–12.66	1,244	205 (16.50)	1.08 (0.87, 1.34)	0.477	1.08 (0.86, 1.36)	0.496
12.66–39.30	1,244	273 (21.90)	1.54 (1.26, 1.89)	< 0.001	1.53 (1.24, 1.90)	< 0.001*
Per IQR = 3.82			1.09 (1.00, 1.19)	0.041	1.09 (1.00, 1.19)	0.055
Whole pregnancy
5.54–9.11	1,264	195 (15.40)	1.00 (1.00, 1.00)	Referent	1.00 (1.00, 1.00)	Referent
9.12–11.06	1,265	175 (13.80)	0.88 (0.71, 1.10)	0.257	0.94 (0.75, 1.18)	0.584
11.07–12.42	1,265	217 (17.20)	1.14 (0.92, 1.40)	0.240	1.16 (0.93, 1.46)	0.186
12.42–29.00	1,265	323 (25.50)	1.88 (1.54, 2.29)	< 0.001	1.92 (1.55, 2.37)	< 0.001*
Per IQR = 3.31			1.38 (1.26, 1.50)	< 0.001	1.41 (1.29, 1.54)	< 0.001*
The last month before delivery
2.26–8.61	1,243	196 (15.80)	1.00 (1.00, 1.00)	Referent	1.00 (1.00, 1.00)	Referent
8.62–10.59	1,242	200 (16.10)	1.03 (0.83, 1.27)	0.820	1.05 (0.84, 1.31)	0.667
10.60–13.09	1,244	253 (20.30)	1.36 (1.11, 1.68)	0.003	1.44 (1.16, 1.79)	0.001*
13.09–52.90	1,244	243 (19.50)	1.30 (1.05, 1.60)	0.014	1.41 (1.13, 1.77)	0.003*
Per IQR = 4.48			1.14 (1.05, 1.23)	0.002	1.13 (1.04, 1.23)	0.004*
Adjusted for maternal smoking status during pregnancy, maternal race/ethnicity, maternal age at delivery, maternal prepregnancy BMI, maternal education level, sex of baby, parity, season of delivery. *Significant after Bonferroni correction for multiple comparisons (*p* < 0.008).						

### Association between Maternal Exposure to PM_2.5_ and IUI

**Figure 2:**
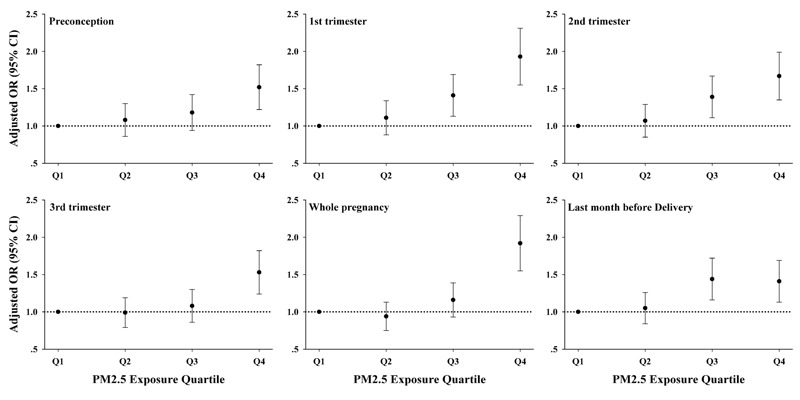
Adjusted ORs quantifying associations between PM_2.5_ exposure and IUI by quartile and period of prepregnancy or pregnancy in 5,059 mothers from 1999 to 2012.

Comparing the highest with the lowest PM_2.5_ exposure quartiles, PM_2.5_ was positively associated with IUI during all exposure periods, including exposure during the 3 months before conception, both before and after adjusting for relevant covariates (Table 2, Figure 2). The effect estimates were similar across different exposure periods, though associations were strongest for PM_2.5_ exposure during the first trimester and over the entire pregnancy (OR = 1.93; 95% CI: 1.55, 2.40 and OR = 1.92; 95% CI: 1.55, 2.37 for the highest versus lowest quartiles during each time period, respectively). When ORs were modeled per IQR increase in PM_2.5_ exposure, the association was significant at the α = 0.008 level for the periods of the first trimester, second trimester, the entire pregnancy, and the last month before delivery.

After adjusting for exposure to PM_2.5_ during the other exposure periods (in addition to all other covariates already in the model), the ORs by quartile and per IQR for the first trimester of pregnancy were consistent with ORs estimated by the single time period model (see Table S2). The OR comparing the fourth and first quartiles of exposure during the first trimester was significant at the 0.008 level (OR = 1.85; 95% CI: 1.37, 2.50 for the multiple time–period model and OR = 1.93; 95% CI: 1.55, 2.40, for the single time–period model). ORs by quartile of exposure during the second trimester were no longer significant after adjusting for other periods of exposure; the OR per IQR was still significant. ORs by quartiles and per IQR were near or below the null for both the period before the pregnancy and the third trimester, after adjusting for exposure during the other three time periods. Exposures to PM_2.5_ during the six time periods examined were moderately to highly correlated (Spearman coefficients 0.43–0.86) (see Table S3). The highest correlations were observed between exposures during the period of the entire pregnancy and each of the three trimesters (Spearman ρ = 0.76–0.86). The correlation between exposures during first and third trimesters was also moderate to high (Spearman ρ = 0.71).

To assess bias due to exposure misclassification among participants located furthest from a monitor, we ran our models in subgroups living within 10 km [*n* = 4,357 (768 cases)] and 4 km [*n* = 1,719 (*n* = 307 cases)] of an ambient PM_2.5_ monitor. Effect estimates in these two subgroups (see Tables S4 and S5) were consistent with results of our main model (Table 2). The association was strongest for exposure during the first trimester among participants within 4 km of a monitor (OR = 2.15; 95% CI: 1.47, 3.14 for the highest versus lowest quartile, compared with OR = 1.93; 95% CI: 1.55, 2.40 for the total study population).

In a stratified analysis to evaluate differences by race/ethnicity, the relationship between maternal exposure to PM_2.5_ and IUI was generally consistent across African-American and non–African-American participants (see Table S6). There were no consistent differences in associations by subgroup. Associations between IUI and PM_2.5_ exposure during trimesters 1 and 2 and the entire pregnancy were also generally robust to stratification by warm and cold months (see Table S7), with a few exceptions. Exposure to PM_2.5_ during preconception during the warm season was not significantly associated with IUI. Conversely, PM_2.5_ during the third trimester during the cold season was not significantly associated with IUI.

In a stratified analysis by two study periods, 1999–2005 and 2006–2012, exposures to PM_2.5_ were generally higher in the early period, during which 3,368 (66.6%) of the participants including 664 (72.3%) of the cases were recruited, compared with the later period. The effect estimates in the early period were consistent with findings of the main analysis (see Table S8). The strongest associations in this stratum were seen for exposure during the first trimester (OR = 2.30; 95% CI: 1.58, 3.34) and over the entire pregnancy (OR = 2.57; 95% CI: 1.75, 3.76). No association was observed in the low exposure period (2006–2012), but it should be noted that the same cut points between exposure quartiles were applied in both strata, and thus there were a low number of subjects in the third and fourth quartiles of exposure during the later period.

## Discussion

### Summary of Results

IUI is a risk factor for preterm birth, low birth weight, and poor respiratory outcomes in early childhood [Gupta et al. 2007; Institute of Medicine (U.S.) Committee on Understanding Premature Birth and Assuring Healthy Outcomes 2007; Kumar et al. 2008; Mestan et al. 2010]. The etiology of these conditions is poorly understood, and inflammation is thought to play a role. Using placenta histopathology and intrapartum fever data from the Boston Birth Cohort, a cohort of mother–infant pairs from an at-risk urban and mostly minority population, we investigated the relationship between exposure to ambient PM_2.5_ just before and during pregnancy and IUI. Comparing the highest with the lowest PM_2.5_ exposure quartiles, the association was significant for all periods of the pregnancy considered. Associations were generally consistent between African-American and non–African-American participants.

The strongest association was observed for periods of the first trimester and the entire pregnancy. This suggests that early pregnancy may be a window of susceptibility to effects of PM_2.5_ on IUI. Moderate to strong positive correlations between exposures during different periods of pregnancy limited our ability to estimate associations that were specific to exposures at different periods of the pregnancy in the single time–point models used in our main analysis. When exposures during multiple time points (i.e, pregnancy periods) were included in the model, the association was still the strongest in the first trimester, supporting the results of the single time–point model. The results of the multiple time–point model should be interpreted with caution due to collinearity in the model resulting from correlation between exposures during different periods.

By treating exposure as a categorical variable (quartiles), we were able to explore the association between maternal PM_2.5_ exposure and IUI without imposing linearity. We did not observe a threshold of exposure-effect at the levels at which our population was exposed. Estimated exposures to PM_2.5_ during the period of the entire pregnancy were below the federal annual standard of 12 μg/m^3^ in 68.61% of the participants included in this analysis. For the first and second trimesters, ORs were significant when comparing quartile 3 to quartile 1, even though quartile 3 included subjects exposed at concentrations < 12 μg/m^3^. The current National Ambient Air Quality Standard for PM_2.5_, last revised in 2013, is based on associated risks in adults including premature mortality and chronic respiratory disease (U.S. EPA 2013). Risks to the fetus were considered in the ruling, but the evidence available at the time of the final ruling was considered to be emerging. Our findings add to a growing body of literature suggesting that pregnant women are a sensitive subpopulation that must be considered during review of the PM_2.5_ standard, because there is increasing evidence that PM_2.5_ exposures near or below the current standard may have adverse effects on the mother and developing fetus.

The annual mean of the daily ambient PM_2.5_ concentration in Boston at the start of the study period was 16.3 μg/m^3^, decreasing to an annual mean of 9.5 μg/m^3^ in 2012 during the study period (U.S. EPA 2015a). These exposure levels are low compared with those in more highly polluted cities inside and outside the United States, such as Fresno, California (44.6 μg/m^3^ in 2012), Dehli, India (153 μg/m^3^ in 2013), and Beijing, China (56 μg/m^3^) (U.S. EPA 2015b; WHO 2014). For this reason, it is remarkable that we observed a significant effect on IUI despite the relatively low level of exposure in this population.

### Comparison with Other Studies

The need for placental tissue histology and clinical data for IUI detection makes study of IUI challenging. The association between air pollution and IUI has not been assessed previously in humans, so we cannot compare our findings to other studies of this exposure and outcome. However, because IUI is a risk factor for preterm birth, we can view our findings in the context of studies of PM_2.5_ exposure during pregnancy and preterm birth. The first trimester has emerged as an exposure window of interest; significant associations between exposure to PM_2.5_ during first trimester and preterm birth have been reported in several studies (Lee et al. 2013; Pereira et al. 2014; Rappazzo et al. 2014).

In addition, exposure to particulate matter early on in pregnancy has been reported to be associated with elevated maternal blood C-reactive protein levels in early pregnancy in studies conducted in the United States and the Netherlands (Lee et al. 2011; van den Hooven et al. 2012a). In the latter study, exposure to particulate matter and NO_2_ were associated with elevated fetal (cord blood) C-reactive protein as well as blood biomarkers consistent with adverse changes in placental growth and function (van den Hooven et al. 2012a, 2012b).

### Strengths and Limitations

Restriction to a single city limits the generalizability of our results, because the proportion of PM_2.5_ from regional and local sources varies by geographic location, which affects the elemental composition of PM at the city level. In Boston, regional sources (i.e., coal burning power plants) are the greatest contributor to PM_2.5_, accounting for about half of PM_2.5_ from all sources (Masri et al. 2015).

Our exposure assessment methodology does not account for differences in exposures due to variation in distance from a stationary monitor. However, > 85% of our population lived within 10 km of a monitor, and exposure estimates for PM_2.5_ concentrations from spatial models do not change substantially at a resolution of < 10 km^2^ due to the homogenous distribution of fine particulate matter over this area (Kloog et al. 2012). The consistency of our results when we ran the analysis in subgroups of subjects within 10 km and 4 km of a monitor also suggests that variation in subject distance from ambient monitors did not bias our exposure estimates.

By adjusting for season in the model, we may have accounted for some confounding by temporally varying factors for which we did not have direct measurements, such as seasonal influenza and respiratory syncytial virus. However, some residual confounding by temporally varying factors may be unaccounted for.

IUI was assessed cross-sectionally in our study and thus does not provide insight into the dynamics of IUI before the time of birth. Due to the invasive nature of available methods for identifying IUI, little is known about whether IUI identified at birth represents recent or chronic IUI, and whether IUI might be present at earlier periods, but not at birth. The strong associations between PM_2.5_ exposure during the first trimester or over the entire pregnancy with IUI suggest that the first trimester may be an important window of exposure for IUI and that chronic exposure may also be important. Data on IUI before birth, if they were available, would help to elucidate the temporal relationship between PM_2.5_ exposure and development of IUI.

Though the placenta histopathology at birth provides a snapshot at one point in time, the use of these data to define IUI is a strength of the study because histopathology captures subclinical and less severe inflammation which may not be detected by other methods such as bacterial culture of amniotic fluid or diagnosis of IUI solely based on fever at parturition. Data on fever were more widely available in the study population than histopathology data. Though fever at parturition is a validated criterion for clinical diagnosis of intrauterine infection (Gibbs et al. 1982), it may be present for reasons other than IUI, or may not be present even when there is histologic evidence of IUI (Goldenberg et al. 2000). As a result, the potential exists for false positives and false negatives among individuals with data on fever but no histopathology data.

The placement of 2,024 (40.0%) participants for whom no income data were available in a missing category is a potential source of bias in our estimates. As the high proportion of participants in the study who qualify for Medicaid (> 90%) reflects, household income is relatively homogeneous among study participants, which may have reduced potential bias due to missing income data.

We also stratified by cool and warm seasons, which serves the purpose of accounting for differences in PM_2.5_ composition during those periods. The findings of the stratified analysis were consistent with the main analysis, and consistent across warm and cold periods, for the three periods for which we saw a strong associations (first trimester, second trimester, and whole pregnancy). This strengthens our confidence that the observed associations between PM_2.5_ exposure and IUI are robust.

A strength of the study was the use of questionnaire data to account for changes in smoking status or residential address during periods of preconception and pregnancy. Despite adjustment for a wide range of covariates including smoking, some residual confounding from unidentified sources may have not been accounted for in our model. Likewise, data on changes in residential address reduced exposure misclassification, though some likely remains due to other factors such as lack of information on exposures at locations other than the residence where participants spent time and the use of a stationary ambient monitor as a surrogate for personal exposure.

## Conclusions

Despite a relatively low level of exposure, our results suggest a monotonic positive relationship relationship between PM_2.5_ exposure during preconception and each trimester of pregnancy and IUI at birth. Our findings suggest that IUI may be a sensitive biomarker for assessing early biological effect of PM_2.5_ exposure on the developing fetus, which may in turn influence subsequent growth, development, and health outcomes.

***Editor’s Note:*** *In the Advance Publication, data points in* *Figure 2* *were located incorrectly on the graphs. The corrected* *Figure 2* *is included in this article and now matches the adjusted odds ratio data in* *Table 2* *. The authors regret this error.*

## Supplemental Material

(561 KB) PDF
