# Supplementary material for: Intrauterine Inflammation and Maternal Exposure to Ambient PM2.5 during Preconception and Specific Periods of Pregnancy: The Boston Birth Cohort
Source: Environ Health Perspect. 2016 Apr 27;124(10):1608–15. doi: 10.1289/EHP243 (PMC5047781; doi:10.1289/EHP243)
Supplement: (561 KB) PDF [file EHP243.s001.acco.pdf]

Environ Health Perspect
DOI: 10.1289/EHP243

Note to readers with disabilities: *EHP* strives to ensure that all journal content is accessible to all readers. However, some figures and Supplemental Material published in *EHP* articles may not conform to 508 standards due to the complexity of the information being presented. If you need assistance accessing journal content, please contact ehp508@niehs.nih.gov. Our staff will work with you to assess and meet your accessibility needs within 3 working days.

Supplemental Material

Intrauterine Inflammation and Maternal Exposure to Ambient $PM_{2.5}$ during Preconception and Specific Periods of Pregnancy: The Boston Birth Cohort

Rebecca Massa Nachman, Guangyun Mao, Xingyou Zhang, Xiumei Hong, Zhu Chen, Claire Sampankanpanich Soria, Huan He, Guoying Wang, Deanna Caruso, Colleen Pearson, Shyam Biswal, Barry Zuckerman, Marsha Wills-Karp, and Xiaobin Wang

Table of Contents

Table S1. Birth-Related Covariates and Characteristics of Subjects Included in or Excluded from the Analysis

Table S2. Associations between Maternal Exposure to Ambient $PM_{2.5}$ (Quartile) and Odds of IUI in the Study Population for Four Pregnancy Periods, Adjusting for the Other Three Periods

Table S3. Spearman Correlation Coefficients for Exposure during Preconception (Pre), Trimester 1 (T1), Trimester 2 (T2), Trimester 3 (T3), the Whole Pregnancy (WP), and the Last Month (LM)

Table S4. Associations between Maternal Exposure to Ambient $PM_{2.5}$ (Quartile) and Odds of IUI (Subjects within 10 km of a Monitor)

Table S5. Associations between Maternal Exposure to Ambient $PM_{2.5}$ (Quartile) and Odds of IUI (Subjects within 4 km of a Monitor)

Table S6. Associations between Maternal Exposure to Ambient $PM_{2.5}$ (Quartile) and Odds of IUI in the Study Population, Stratified by African American or non-African American

Table S7. Associations between Maternal Exposure to Ambient $PM_{2.5}$ (Quartile) and Odds of IUI in the Study Population, Stratified by Warm (May-Sept) and Cold (Oct-April) Season

Table S8. Associations between Maternal Exposure to Ambient $PM_{2.5}$ (Quartile) and Odds of IUI in the Study Population, Stratified by Study Periods: 1999-2005 (high $PM_{2.5}$ levels) and 2006-2012 (low $PM_{2.5}$ levels).

Figure S1. Flow Chart of Subjects Enrolled in the Boston Birth Cohort Included in or Excluded from the Current Analysis

Figure S2. Mean Ambient $PM_{2.5}$ Exposure over Time in a Subset of 5,059 Mothers in the Boston Birth Cohort from 1999-2012

Table S1. Birth-Related Covariates and Characteristics for Subjects Included in and Subjects Excluded from the Analysis

**Characteristics**	**Excluded** ***(N=2777)***	**Included** ***(N=5059)***	***p***
**Maternal characteristics**			
Age at delivery, years			0.017
<20	272(9.79)	568(11.23)	
20-24	664(23.91)	1286(25.42)	
25-29	751(27.04)	1269(25.08)	
30-34	635(22.87)	1116(22.06)	
35+	452(16.28)	820(16.21)	
Missing	3(0.11)	0(0.00)	
Pre-pregnancy BMI, kg/m^2			<0.001
<18.5	110(3.96)	231(4.57)	
18.5-24.9	1187(42.74)	2373(46.91)	
25.0-29.9	718(25.86)	1392(27.52)	
30.0±	529(19.05)	942(18.62)	
Missing	233(8.39)	121(2.39)	
Race/Ethnic			<0.001
Hispanic	816(29.38)	1398(27.63)	
White	335(12.06)	569(11.25)	
African/African American	1201(43.25)	2488(49.18)	
Others	353(12.71)	597(11.80)	
Missing	72(2.59)	7(0.14)	
Smoking during pregnancy			<0.001
Never smoking before	2222(80.10)	3971(78.49)	
Quit smoking	184(6.63)	359(7.10)	
Continue smoking	299(10.77)	691(13.66)	
Missing	69(2.48)	38(0.75)	
Education category			<0.001
Middle school or below	957(34.46)	1697(33.54)	
High school	905(32.59)	1662(32.85)	
College or above	833(30.00)	1652(32.65)	
Missing	82(2.95)	48(0.95)	
Household income per year			<0.001
<$25,000	818(29.49)	2198(43.45)	
$25,000+	475(17.12)	837(16.54)	
Missing	1481(53.39)	2024(40.01)	
Place of birth			<0.001
Non-USA	1724(62.08)	3095(61.18)	
USA	960(34.57)	1936(38.27)	
Missing	93(3.35)	28(0.55)	

Season at delivery			0.005
Spring (Mar. to May)	691(24.88)	1133(22.40)	
Summer (June to Aug.)	731(26.32)	1256(24.83)	
Autumn (Sept. to Nov.)	706(25.42)	1346(26.61)	
Winter (Dec. to Feb.)	649(23.37)	1324(26.17)	
Parity			<0.001
0	1149(41.38)	2139(42.28)	
1+	1561(56.21)	2916(57.64)	
Missing	67(2.41)	4(0.08)	
**Children characteristics**			
Preterm birth			<0.001
No	2214(79.73)	3492(69.03)	
Yes	497(17.90)	1567(30.97)	
Missing	66(2.38)	0(0.00)	
Gender			
Girl	1361(49.01)	2565(50.70)	
Boy	1416(50.99)	2492(49.26)	
Missing	0(0.00)	2(0.04)	
Gestational age, weeks [a]	39.14(37.71,40.29)	38.57(36.29,40.00)	<0.001

[a] Data did not meet normal distribution and are described as median (IQR), and Mann-Whitney U test was applied to compare the difference between two groups.

Table S2. Associations between Maternal Exposure to Ambient $PM_{2.5}$ (Quartile) and Odds of IUI in the Study Population for Four Pregnancy Periods, Adjusting for the Other Three Periods

$PM_{2.5}$, μg/m^3	N	Cases (%)	*Crude*		*Adjusted*	
			OR(95%CI)	*p*	*OR(95%CI)*	*p*
Preconception						
1.2-9.06	1212	182(15.00)	1.00(1.00,1.00)	Ref.	1.00(1.00,1.00)	Ref.
9.07-10.85	1212	194(16.00)	1.08(0.87,1.34)	0.501	0.88(0.68,1.14)	0.327
10.86-12.73	1213	210(17.30)	1.19(0.95,1.47)	0.125	0.88(0.66,1.17)	0.376
12.73-29.00	1212	255(21.00)	1.51(1.22,1.86)	<0.001	1.06(0.78,1.43)	0.721
Per IQR=3.67			1.05(0.97,1.13)	0.239	0.91(0.83,0.99)	0.026
1st trimester						
4.16-8.99	1239	171(13.80)	1.00(1.00,1.00)	Ref.	1.00(1.00,1.00)	Ref.
9.00-10.95	1228	180(14.70)	1.07(0.86,1.35)	0.543	1.12(0.86,1.45)	0.398
10.96-12.72	1234	225(18.20)	1.39(1.12,1.73)	0.003	1.45(1.08,1.93)	0.012
12.72-27.72	1233	288(23.40)	1.90(1.55,2.35)	<0.001	1.85(1.37,2.50)	<0.001
Per IQR=3.73			1.27(1.17,1.39)	<0.001	1.26(1.14,1.40)	<0.001
2nd trimester						
4.44-8.97	1249	183(14.70)	1.00(1.00,1.00)	Ref.	1.00(1.00,1.00)	Ref.
8.98-10.96	1250	194(15.50)	1.07(0.86,1.33)	0.544	1.00(0.77,1.30)	0.994
10.97-12.58	1246	238(19.10)	1.38(1.11,1.70)	0.003	1.24(0.92,1.66)	0.157
12.58-52.90	1251	280(22.40)	1.68(1.37,2.06)	<0.001	1.27(0.93,1.74)	0.134
Per IQR=3.61			1.30(1.20,1.42)	<0.001	1.29(1.18,1.42)	<0.001
3rd trimester						
3.20-8.85	1244	192(15.40)	1.00(1.00,1.00)	Ref.	1.00(1.00,1.00)	Ref.
8.86-10.85	1244	187(15.00)	0.97(0.78,1.21)	0.780	0.76(0.59,0.99)	0.041
10.86-12.66	1244	205(16.50)	1.08(0.87,1.34)	0.477	0.65(0.49,0.87)	0.003
12.66-39.30	1244	273(21.90)	1.54(1.26,1.89)	<0.001	0.91(0.67,1.22)	0.517
Per IQR=3.82			1.09(1.00,1.19)	0.041	0.93(0.84,1.03)	0.159

IQR indicates interquartile range; Adjusted for maternal smoking status during pregnancy, maternal race/ethnicity, maternal age at delivery, maternal pre-pregnancy body mass index, maternal education level, sex of baby, parity, season of delivery and house hold income per year.

Table S3. Spearman Correlation Coefficients for Exposure during Preconception (Pre), Trimester 1 (T1), Trimester 2 (T2), Trimester 3 (T3), the Whole Pregnancy (WP), and the Last Month (LM)

Pregnancy Period	Pre	T1	T2	T3	WP	LM
Pre	1.00	0.46	0.73	0.43	0.66	0.36
T1		1.00	0.47	0.71	0.86	0.56
T2			1.00	0.47	0.76	0.41
T3				1.00	0.86	0.78
WP					1.00	0.70
LM						1.00

Table S4. Associations between Maternal Exposure to Ambient $PM_{2.5}$ (Quartile) and Odds of Intrauterine Inflammation (Subjects within 10 km of a Monitor)

$PM_{2.5}$, μg/m^3	N	Cases (%)	*Crude*		*Adjusted*	
			OR(95%CI)	*p*	*OR(95%CI)*	*p*
Preconception						
1.20-9.21	1045	163(15.60)	1.00(1.00,1.00)	Ref.	1.00(1.00,1.00)	Ref.
9.22-10.95	1046	158(15.10)	0.96(0.76,1.22)	0.755	0.96(0.75,1.23)	0.735
10.96-12.75	1046	177(16.90)	1.10(0.87,1.39)	0.412	1.08(0.84,1.37)	0.559
12.76-29.00	1046	213(20.40)	1.38(1.11,1.73)	0.005	1.38(1.09,1.75)	0.008
Per IQR=3.54			1.02(0.94,1.11)	0.622	1.03(0.94,1.12)	0.565
1st trimester						
4.17-9.07	1064	145(13.60)	1.00(1.00,1.00)	Ref.	1.00(1.00,1.00)	Ref.
9.08-11.06	1063	161(15.10)	1.13(0.89,1.44)	0.319	1.16(0.90,1.49)	0.243
11.07-12.80	1065	184(17.30)	1.32(1.05,1.68)	0.020	1.33(1.04,1.70)	0.026
12.81-27.72	1064	241(22.70)	1.86(1.48,2.33)	<0.001	1.87(1.48,2.38)	<0.001
Per IQR=3.72			1.24(1.13,1.36)	<0.001	1.26(1.15,1.39)	<0.001
2nd trimester						
4.44-9.12	1077	160(14.90)	1.00(1.00,1.00)	Ref.	1.00(1.00,1.00)	Ref.
9.13-11.01	1073	164(15.30)	1.03(0.82,1.31)	0.781	1.02(0.80,1.31)	0.862
11.02-12.55	1077	203(18.80)	1.33(1.06,1.67)	0.014	1.35(1.06,1.71)	0.016
12.56-52.90	1076	229(21.30)	1.55(1.24,1.94)	<0.001	1.53(1.21,1.94)	<0.001
Per IQR=3.44			1.24(1.14,1.36)	<0.001	1.25(1.14,1.37)	<0.001
3rd trimester						
3.20-9.01	1073	163(15.20)	1.00(1.00,1.00)	Ref.	1.00(1.00,1.00)	Ref.
9.02-10.93	1074	162(15.10)	0.99(0.78,1.26)	0.945	1.04(0.81,1.33)	0.751
10.94-12.68	1074	172(16.00)	1.07(0.84,1.34)	0.599	1.07(0.84,1.37)	0.598
12.69-39.30	1073	233(21.70)	1.55(1.24,1.93)	<0.001	1.56(1.23,1.97)	<0.001
Per IQR=3.67			1.10(1.00,1.21)	0.059	1.10(1.00,1.22)	0.054
Whole pregnancy						
5.54-9.28	1089	171(15.70)	1.00(1.00,1.00)	Ref.	1.00(1.00,1.00)	Ref.
9.29-11.11	1089	144(13.20)	0.82(0.64,1.04)	0.100	0.88(0.69,1.13)	0.320
11.12-12.36	1091	185(17.00)	1.10(0.87,1.38)	0.428	1.12(0.88,1.43)	0.358
12.37-29.00	1088	269(24.70)	1.76(1.42,2.18)	<0.001	1.81(1.44,2.27)	<0.001
Per IQR=3.08			1.35(1.23,1.48)	<0.001	1.39(1.26,1.53)	<0.001
The last month before delivery						
2.26-8.72	1070	160(15.00)	1.00(1.00,1.00)	Ref.	1.00(1.00,1.00)	Ref.
8.73-10.73	1071	174(16.20)	1.10(0.87,1.39)	0.410	1.15(0.90,1.46)	0.262
10.74-13.25	1069	223(20.90)	1.50(1.20,1.88)	<0.001	1.59(1.26,2.02)	<0.001
13.26-52.90	1072	197(18.40)	1.28(1.02,1.61)	0.034	1.38(1.07,1.77)	0.012
Per IQR=4.53			1.13(1.04,1.24)	0.006	1.13(1.03,1.24)	0.009

IQR indicates interquartile range; Adjusted for maternal smoking status during pregnancy, maternal race/ethnicity, maternal age at delivery, maternal pre-pregnancy body mass index, maternal education level, sex of baby, parity, season of delivery and house hold income per year.

Table S5 Associations between Maternal Exposure to Ambient $PM_{2.5}$ (Quartile) and Odds of IUI (Subjects within 4 km of a Monitor)

$PM_{2.5}$, μg/m^3	N	Cases (%)	*Crude*		*Adjusted*	
			OR(95%CI)	*p*	*OR(95%CI)*	*p*
Preconception						
1.20-8.95	413	63(15.30)	1.00(1.00,1.00)	Ref.	1.00(1.00,1.00)	Ref.
8.96-10.70	414	68(16.40)	1.09(0.75,1.59)	0.645	1.13(0.77,1.67)	0.525
10.71-12.47	414	64(15.50)	1.02(0.70,1.48)	0.935	1.06(0.71,1.58)	0.771
12.48-29.00	413	94(22.80)	1.64(1.15,2.33)	0.006	1.79(1.22,2.61)	0.003
Per IQR=3.51			1.10(0.96,1.26)	0.185	1.11(0.96,1.28)	0.170
1st trimester						
4.44-8.88	420	60(14.30)	1.00(1.00,1.00)	Ref.	1.00(1.00,1.00)	Ref.
8.89-10.65	420	61(14.50)	1.02(0.69,1.50)	0.922	1.07(0.71,1.59)	0.753
10.66-12.72	420	71(16.90)	1.22(0.84,1.77)	0.296	1.31(0.88,1.96)	0.180
12.73-27.72	420	102(24.30)	1.93(1.35,2.74)	<0.001	2.15(1.47,3.14)	<0.001
Per IQR=3.84			1.27(1.10,1.47)	0.001	1.33(1.14,1.55)	<0.001
2nd trimester						
4.44-8.96	424	69(16.30)	1.00(1.00,1.00)	Ref.	1.00(1.00,1.00)	Ref.
8.97-10.76	424	64(15.10)	0.92(0.63,1.33)	0.637	0.92(0.63,1.35)	0.675
10.77-12.58	425	71(16.70)	1.03(0.72,1.48)	0.865	1.13(0.77,1.67)	0.537
12.59-52.90	424	98(23.10)	1.55(1.10,2.18)	0.013	1.65(1.14,2.40)	0.009
Per IQR=3.61			1.14(0.99,1.30)	0.064	1.17(1.01,1.35)	0.032
3rd trimester						
4.20-8.79	424	69(16.30)	1.00(1.00,1.00)	Ref.	1.00(1.00,1.00)	Ref.
8.80-10.72	424	63(14.90)	0.90(0.62,1.30)	0.570	0.97(0.66,1.44)	0.890
10.73-12.62	425	70(16.50)	1.01(0.71,1.46)	0.938	1.11(0.75,1.64)	0.601
12.63-39.30	424	93(21.90)	1.45(1.02,2.04)	0.037	1.63(1.12,2.36)	0.011
Per IQR=3.83			1.12(0.97,1.29)	0.138	1.18(1.02,1.37)	0.031
Whole pregnancy						
5.54-9.15	429	70(16.30)	1.00(1.00,1.00)	Ref.	1.00(1.00,1.00)	Ref.
9.16-10.77	430	54(12.60)	0.74(0.50,1.08)	0.118	0.89(0.59,1.34)	0.570
10.78-12.32	430	83(19.30)	1.23(0.86,1.74)	0.253	1.47(1.00,2.16)	0.049
12.33-27.71	430	101(23.50)	1.57(1.12,2.21)	0.009	1.84(1.26,2.68)	0.002
Per IQR=3.17			1.35(1.16,1.57)	<0.001	1.46(1.25,1.71)	<0.001
The last month before delivery						
2.26-8.60	420	68(16.20)	1.00(1.00,1.00)	Ref.	1.00(1.00,1.00)	Ref.
8.61-10.60	421	68(16.20)	1.00(0.69,1.44)	0.988	1.08(0.73,1.59)	0.694
10.61-13.22	421	92(21.90)	1.45(1.02,2.05)	0.037	1.73(1.18,2.53)	0.005
13.23-52.90	420	72(17.10)	1.07(0.75,1.54)	0.711	1.41(0.94,2.12)	0.097
Per IQR=4.60			1.09(0.94,1.25)	0.248	1.13(0.98,1.30)	0.105

IQR indicates interquartile range; Adjusted for maternal smoking status during pregnancy, maternal race/ethnicity, maternal age at delivery, maternal pre-pregnancy body mass index, maternal education level, sex of baby, parity, season of delivery and house hold income per year.

Table S6. Associations between Maternal Exposure to Ambient $PM_{2.5}$ (Quartile) and Odds of IUI in the Study Population, Stratified by African American or Non-African American

African American					*Non-African American*				
$PM_{2.5}$, μg/m^3	N	Cases (%)	*Adjusted OR (95% CI)*	*p*	$PM_{2.5}$, μg/m^3	N	Cases (%)	*Adjusted OR (95% CI)*	*p*
Preconception									
1.20-9.36	614	85(13.80)	1.00(1.00,1.00)	Ref.	4.53-9.78	596	98(16.40)	1.00(1.00,1.00)	Ref.
9.37-11.12	614	91(14.80)	1.10(0.79,1.52)	0.586	9.79-11.54	598	103(17.20)	1.00(0.73,1.38)	0.985
11.13-13.02	614	97(15.80)	1.22(0.88,1.70)	0.230	11.55-13.14	597	113(18.90)	1.18(0.86,1.61)	0.309
13.03-29.00	614	128(20.80)	1.61(1.17,2.22)	0.003	13.15-26.04	597	125(20.90)	1.39(1.02,1.89)	0.036
Per IQR=3.65			1.04(0.93,1.16)	0.518	Per IQR=3.36			1.05(0.94,1.18)	0.364
1st trimester									
4.17-9.26	624	84(13.50)	1.00(1.00,1.00)	Ref.	4.63-9.87	607	93(15.30)	1.00(1.00,1.00)	Ref.
9.27-11.19	625	86(13.80)	1.06(0.77,1.48)	0.710	9.88-11.60	609	96(15.80)	1.08(0.78,1.49)	0.634
11.20-13.07	625	94(15.00)	1.16(0.83,1.62)	0.391	11.61-13.14	607	125(20.60)	1.47(1.08,2.01)	0.014
13.08-27.72	625	144(23.00)	1.96(1.43,2.69)	<0.001	13.15-21.81	607	141(23.20)	1.74(1.29,2.35)	<0.001
Per IQR=3.81			1.24(1.10,1.41)	0.001	Per IQR=3.28			1.35(1.18,1.53)	<0.001
2nd trimester									
4.71-9.17	633	82(13.00)	1.00(1.00,1.00)	Ref.	4.57-9.70	613	97(15.80)	1.00(1.00,1.00)	Ref.
9.18-11.12	634	102(16.10)	1.28(0.92,1.76)	0.141	9.71-11.53	614	101(16.40)	1.04(0.76,1.43)	0.805
11.13-12.73	634	113(17.80)	1.51(1.09,2.09)	0.013	11.54-12.86	614	117(19.10)	1.26(0.93,1.72)	0.142
12.74-52.90	634	127(20.00)	1.66(1.20,2.29)	0.002	12.87-29.00	613	154(25.10)	1.81(1.34,2.44)	<0.001
Per IQR=3.55			1.22(1.08,1.37)	0.001	Per IQR=3.16			1.38(1.22,1.56)	<0.001
3rd trimester									
3.20-9.16	634	98(15.50)	1.00(1.00,1.00)	Ref.	3.79-9.54	608	98(16.10)	1.00(1.00,1.00)	Ref.
9.17-11.16	634	81(12.80)	0.81(0.58,1.12)	0.198	9.55-11.38	608	101(16.60)	1.03(0.75,1.41)	0.868
11.17-12.88	634	96(15.10)	1.00(0.72,1.38)	0.988	11.39-12.96	609	111(18.20)	1.15(0.84,1.58)	0.374
12.89-39.30	634	145(22.90)	1.66(1.22,2.25)	0.001	12.97-30.25	608	125(20.60)	1.27(0.93,1.72)	0.132
Per IQR=3.71					Per IQR=3.42			0.99(0.88,1.13)	0.908

	African American					Non-African American			
$PM_{2.5}$, μg/m^3	N	Cases (%)	*Adjusted OR (95% CI)*	*p*	$PM_{2.5}$, μg/m^3	N	Cases (%)	*Adjusted OR (95% CI)*	*p*
Whole pregnancy									
5.54-9.41	641	96(15.00)	1.00(1.00,1.00)	Ref.	5.86-9.89	622	98(15.80)	1.00(1.00,1.00)	Ref.
9.42-11.18	641	78(12.20)	0.84(0.60,1.17)	0.301	9.90-11.50	622	101(16.20)	1.07(0.78,1.46)	0.688
11.19-12.60	641	102(15.90)	1.15(0.83,1.59)	0.404	11.51-12.54	622	109(17.50)	1.19(0.87,1.63)	0.278
12.61-29.00	641	158(24.60)	1.93(1.42,2.63)	<0.001	12.55-27.71	622	166(26.70)	2.01(1.49,2.70)	<0.001
Per IQR=3.18			1.47(1.28,1.68)	<0.001	Per IQR=2.64			1.33(1.18,1.51)	<0.001
The last month before delivery									
2.26-8.85	630	94(14.90)	1.00(1.00,1.00)	Ref.	2.26-9.06	612	102(16.70)	1.00(1.00,1.00)	Ref.
8.86-10.97	629	92(14.60)	1.01(0.73,1.40)	0.955	9.07-11.03	611	109(17.80)	1.11(0.82,1.51)	0.490
10.98-13.46	631	112(17.70)	1.37(0.99,1.89)	0.055	11.04-13.50	612	138(22.50)	1.48(1.10,2.01)	0.010
13.47-52.90	630	126(20.00)	1.72(1.24,2.39)	0.001	13.51-32.80	611	117(19.10)	1.17(0.86,1.61)	0.322
Per IQR=4.61			1.17(1.04,1.31)	0.011	Per IQR=4.43			1.09(0.96,1.23)	0.178

IQR indicates interquartile range; Adjusted for maternal smoking status during pregnancy, maternal race/ethnicity, maternal age at delivery, maternal pre-pregnancy body mass index, maternal education level, sex of baby, parity, season of delivery and house hold income per year.

Table S7. Associations between Maternal Exposure to Ambient $PM_{2.5}$ (Quartile) and Odds of IUI in the Study Population, Stratified by Warm (May-Sept) and Cold (Oct-April) Season

Cold					*Warm*				
$PM_{2.5}$, μg/m^3	N	Cases (%)	*Adjusted OR (95% CI)*	*p*	$PM_{2.5}$, μg/m^3	N	Cases (%)	*Adjusted OR (95% CI)*	*p*
Preconception									
1.20-9.11	714	106(14.80)	1.00(1.00,1.00)	Ref.	4.77-9.23	498	75(15.10)	1.00(1.00,1.00)	Ref.
9.11-11.14	715	117(16.40)	1.18(0.87,1.58)	0.287	9.23-10.76	497	78(15.70)	1.04(0.73,1.49)	0.814
11.14-12.8	715	119(16.60)	1.21(0.89,1.63)	0.221	10.76-12.73	498	91(18.30)	1.29(0.91,1.84)	0.154
12.81-27.71	715	167(23.40)	1.83(1.37,2.44)	<0.001	12.73-29.00	497	88(17.70)	1.29(0.89,1.86)	0.174
Per IQR=3.69			1.18(1.07,1.31)	0.001	Per IQR=3.50			0.89(0.78,1.01)	0.076
1st trimester									
4.17-9.1	719	105(14.60)	1.00(1.00,1.00)	Ref.	4.98-9.02	514	69(13.40)	1.00(1.00,1.00)	Ref.
9.1-11.15	719	117(16.30)	1.18(0.87,1.58)	0.288	9.03-10.86	514	66(12.80)	0.98(0.67,1.42)	0.904
11.15-12.73	720	129(17.90)	1.35(1.00,1.81)	0.049	10.86-12.86	516	90(17.40)	1.29(0.90,1.85)	0.170
12.74-21.1	719	161(22.40)	1.75(1.31,2.34)	<0.001	12.86-27.72	513	127(24.80)	2.05(1.45,2.91)	<0.001
Per IQR=3.63			1.27(1.12,1.43)	<0.001	Per IQR=3.83			1.32(1.15,1.51)	<0.001
2nd trimester									
4.57-9.2	725	116(16.00)	1.00(1.00,1.00)	Ref.	4.44-9.01	524	70(13.40)	1.00(1.00,1.00)	Ref.
9.2-10.84	723	101(14.00)	0.90(0.66,1.21)	0.472	9.02-11.36	525	91(17.30)	1.35(0.95,1.92)	0.096
10.84-12.41	724	142(19.60)	1.40(1.05,1.87)	0.024	11.36-12.8	526	96(18.30)	1.43(1.00,2.03)	0.050
12.41-29.00	725	165(22.80)	1.67(1.25,2.23)	0.001	12.8-52.9	524	114(21.80)	1.83(1.29,2.59)	0.001
Per IQR=3.21			1.32(1.17,1.48)	<0.001	Per IQR=3.78			1.32(1.16,1.50)	<0.001
3rd trimester									
3.2-8.76	725	122(16.80)	1.00(1.00,1.00)	Ref.	4.53-9.24	518	69(13.30)	1.00(1.00,1.00)	Ref.
8.76-10.69	726	113(15.60)	0.92(0.69,1.23)	0.589	9.24-11.17	519	72(13.90)	1.00(0.69,1.45)	0.987
10.69-12.53	726	121(16.70)	0.98(0.73,1.31)	0.891	11.17-12.92	519	91(17.50)	1.32(0.92,1.88)	0.133
12.53-39.3	725	148(20.40)	1.26(0.95,1.67)	0.116	12.93-26.49	518	121(23.40)	1.85(1.30,2.63)	0.001
Per IQR=3.77			1.02(0.91,1.15)	0.691	Per IQR=3.68			1.22(1.05,1.42)	0.008

Cold					*Warm*				
$PM_{2.5}$, μg/m^3	N	Cases (%)	*Adjusted OR (95% CI)*	*p*	$PM_{2.5}$, μg/m^3	N	Cases (%)	*Adjusted OR (95% CI)*	*p*
Whole pregnancy									
5.67-9.16	738	122(16.50)	1.00(1.00,1.00)	Ref.	5.54-9.26	526	73(13.90)	1.00(1.00,1.00)	Ref.
9.17-11.06	738	108(14.60)	0.96(0.71,1.29)	0.762	9.27-11.21	527	66(12.50)	0.90(0.62,1.30)	0.584
11.06-12.38	739	116(15.70)	1.00(0.74,1.34)	0.980	11.21-12.32	526	101(19.20)	1.44(1.01,2.04)	0.042
12.39-29.00	738	192(26.00)	1.86(1.41,2.46)	<0.001	12.32-16.83	527	132(25.00)	2.02(1.44,2.84)	<0.001
Per IQR=3.22			1.36(1.21,1.53)	<0.001	Per IQR=3.05			1.47(1.27,1.69)	<0.001
The last month before delivery									
2.26-8.55	721	127(17.60)	1.00(1.00,1.00)	Ref.	3.19-9.05	522	71(13.60)	1.00(1.00,1.00)	Ref.
8.56-10.36	721	112(15.50)	0.90(0.67,1.20)	0.464	9.06-11.19	522	94(18.00)	1.31(0.92,1.86)	0.129
10.36-12.74	721	142(19.70)	1.20(0.90,1.59)	0.209	11.19-13.74	523	102(19.50)	1.56(1.09,2.23)	0.015
12.74-52.90	721	144(20.00)	1.26(0.94,1.69)	0.125	13.74-23.34	522	100(19.20)	1.51(1.05,2.16)	0.027
Per IQR=4.18			1.14(1.03,1.27)	0.015	Per IQR=4.68			1.14(0.99,1.32)	0.072

IQR indicates interquartile range; Adjusted for maternal smoking status during pregnancy, maternal race/ethnicity, maternal age at delivery, maternal pre-pregnancy body mass index, maternal education level, sex of baby, parity, season of delivery and house hold income per year.

Table S8. Associations between Maternal Exposure to Ambient PM2.5 (Quartile) and Odds of IUI, Stratified by Study Periods: 1999-2005 (High $PM_{2.5}$ Levels) and 2006-2012 (Low $PM_{2.5}$ Levels).

1999-2005					*2006-2012*				
$PM_{2.5}$, μg/m^3	N	Cases (%)	*Adjusted OR (95% CI)*	*p*	$PM_{2.5}$, μg/m^3	N	Cases (%)	*Adjusted OR (95% CI)*	*p*
Preconception									
1.2-9.06	236	40(16.90)	1.00(1.00,1.00)	Ref.	1.2-9.06	976	142(14.50)	1.00(1.00,1.00)	Ref.
9.07-10.85	680	118(17.40)	1.03(0.69,1.54)	0.884	9.07-10.85	532	76(14.30)	1.02(0.75,1.39)	0.900
10.86-12.73	1054	185(17.60)	1.03(0.70,1.51)	0.877	10.86-12.73	159	25(15.70)	1.09(0.67,1.76)	0.741
12.73-29.00	1188	252(21.20)	1.31(0.90,1.90)	0.163	>12.73	24	3(12.50)	0.82(0.23,2.96)	0.761
Per IQR=3.67	3158		0.96(0.88,1.05)	0.396	Per IQR=3.67	1691		1.03(0.80,1.34)	0.793
1st trimester									
4.16-8.99	304	37(12.20)	1.00(1.00,1.00)	Ref.	4.16-8.99	935	134(14.30)	1.00(1.00,1.00)	Ref.
9.00-10.95	660	94(14.20)	1.29(0.85,1.95)	0.228	9.00-10.95	568	86(15.10)	1.06(0.78,1.44)	0.701
10.96-12.72	1074	203(18.90)	1.74(1.19,2.54)	0.005	10.96-12.72	160	22(13.80)	0.92(0.55,1.53)	0.735
12.72-27.72	1205	284(23.60)	2.30(1.58,3.34)	<0.001	>12.72	28	4(14.30)	1.05(0.34,3.20)	0.933
Per IQR=3.73	3243		1.28(1.15,1.43)	<0.001	Per IQR=3.73	1691		0.94(0.72,1.24)	0.664
2nd trimester									
4.44-8.97	311	43(13.80)	1.00(1.00,1.00)	Ref.	4.44-8.97	938	140(14.90)	1.00(1.00,1.00)	Ref.
8.98-10.96	729	126(17.30)	1.30(0.89,1.90)	0.183	8.98-10.96	521	68(13.10)	0.86(0.62,1.18)	0.348
10.97-12.58	1063	210(19.80)	1.55(1.08,2.23)	0.017	10.97-12.58	183	28(15.30)	1.04(0.65,1.65)	0.868
12.58-52.90	1202	270(22.50)	1.83(1.28,2.61)	0.001	>12.58	49	10(20.40)	1.33(0.62,2.83)	0.462
Per IQR=3.61	3305		1.28(1.15,1.43)	<0.001	Per IQR=3.61	1691		1.06(0.83,1.36)	0.633
3rd trimester									
3.20-8.85	388	59(15.20)	1.00(1.00,1.00)	Ref.	3.20-8.85	856	133(15.50)	1.00(1.00,1.00)	Ref.
8.86-10.85	673	112(16.60)	1.13(0.80,1.61)	0.486	8.86-10.85	571	75(13.10)	0.86(0.62,1.18)	0.353
10.86-12.66	1044	187(17.90)	1.22(0.88,1.69)	0.235	10.86-12.66	200	18(9.00)	0.55(0.32,0.95)	0.031
12.66-39.30	1201	263(21.90)	1.56(1.14,2.13)	0.006	>12.66	43	10(23.30)	2.15(0.97,4.75)	0.059
Per IQR=3.82	3306		1.03(0.93,1.15)	0.561	Per IQR=3.82	1670		0.83(0.63,1.09)	0.185

	1999-2005					2006-2012			
$PM_{2.5}$, μg/m^3	N	Cases (%)	*Adjusted OR (95% CI)*	*p*	$PM_{2.5}$, μg/m^3	N	Cases (%)	*Adjusted OR (95% CI)*	*p*
Whole pregnancy									
5.54-9.11	286	35(12.20)	1.00(1.00,1.00)	Ref.	5.54-9.11	978	160(16.40)	1.00(1.00,1.00)	Ref.
9.12-11.06	590	94(15.90)	1.47(0.96,2.24)	0.076	9.12-11.06	675	81(12.00)	0.72(0.53,0.98)	0.035
11.07-12.42	1227	212(17.30)	1.59(1.07,2.34)	0.020	11.07-12.42	38	5(13.20)	0.67(0.25,1.83)	0.436
12.42-29.00	1265	323(25.50)	2.57(1.75,3.76)	<0.001	>12.42	0	0		
Per IQR=3.31	3368		1.47(1.31,1.65)	<0.001	Per IQR=3.31	1691		0.86(0.65,1.16)	0.328
The last month before delivery									
2.26-8.61	483	70(14.50)	1.00(1.00,1.00)	Ref.	2.26-8.61	760	126(16.60)	1.00(1.00,1.00)	Ref.
8.62-10.59	710	129(18.20)	1.35(0.97,1.86)	0.071	8.62-10.59	532	71(13.30)	0.81(0.58,1.13)	0.214
10.60-13.09	974	222(22.80)	1.82(1.34,2.46)	<0.001	10.60-13.09	270	31(11.50)	0.72(0.45,1.14)	0.161
13.09-52.90	1132	230(20.30)	1.66(1.21,2.26)	0.001	>13.09	112	13(11.60)	0.74(0.38,1.45)	0.380
Per IQR=4.48	3299		1.14(1.03,1.25)	0.012	Per IQR=4.48	1674		0.88(0.67,1.16)	0.381

IQR indicates interquartile range; Adjusted for maternal smoking status during pregnancy, maternal race/ethnicity, maternal age at delivery, maternal pre-pregnancy body mass index, maternal education level, sex of baby, parity, season of delivery and house hold income per year.

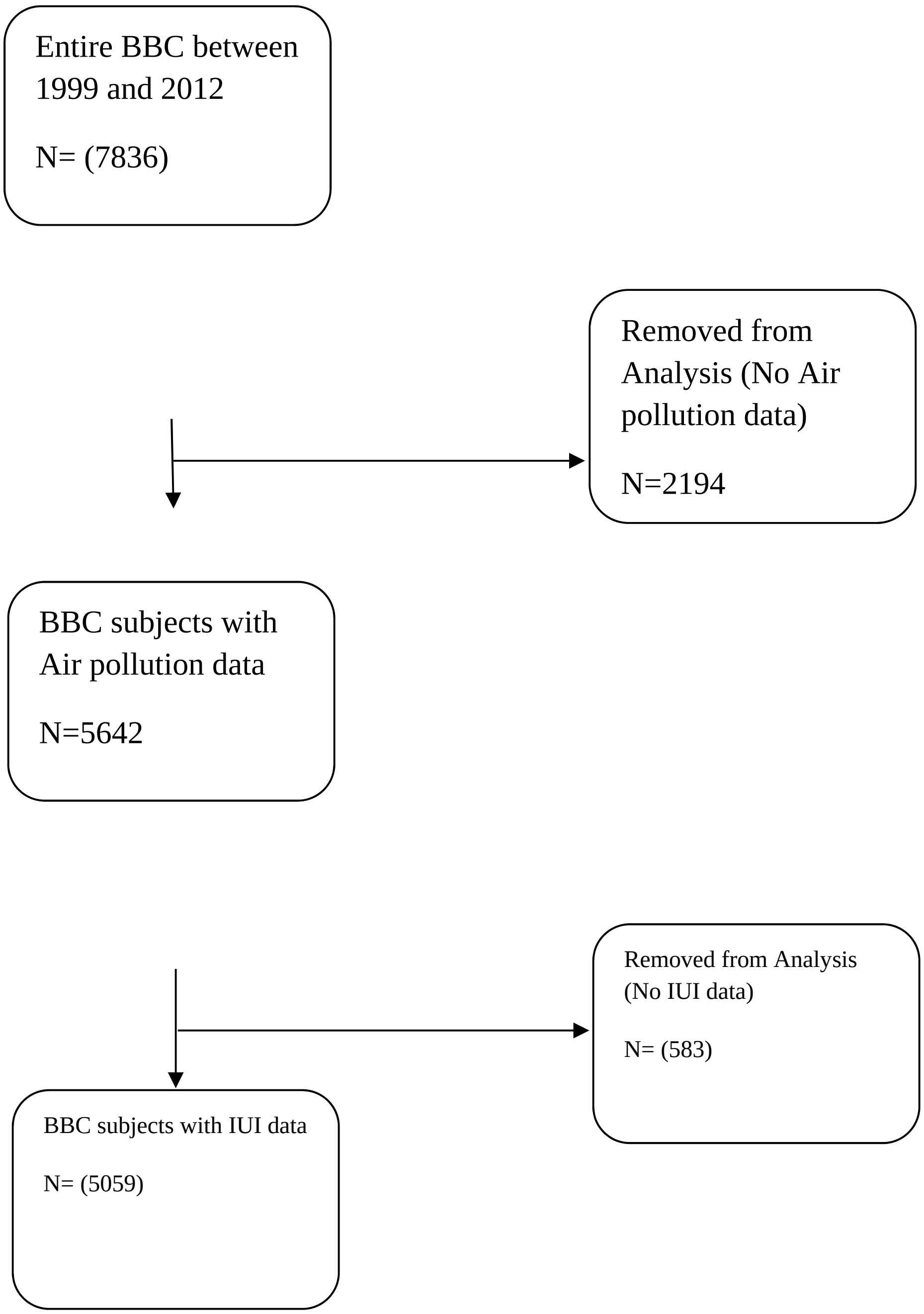

Figure S1. Flow Chart of Subjects Enrolled in the Boston Birth Cohort Included in or Excluded from the Current Analysis

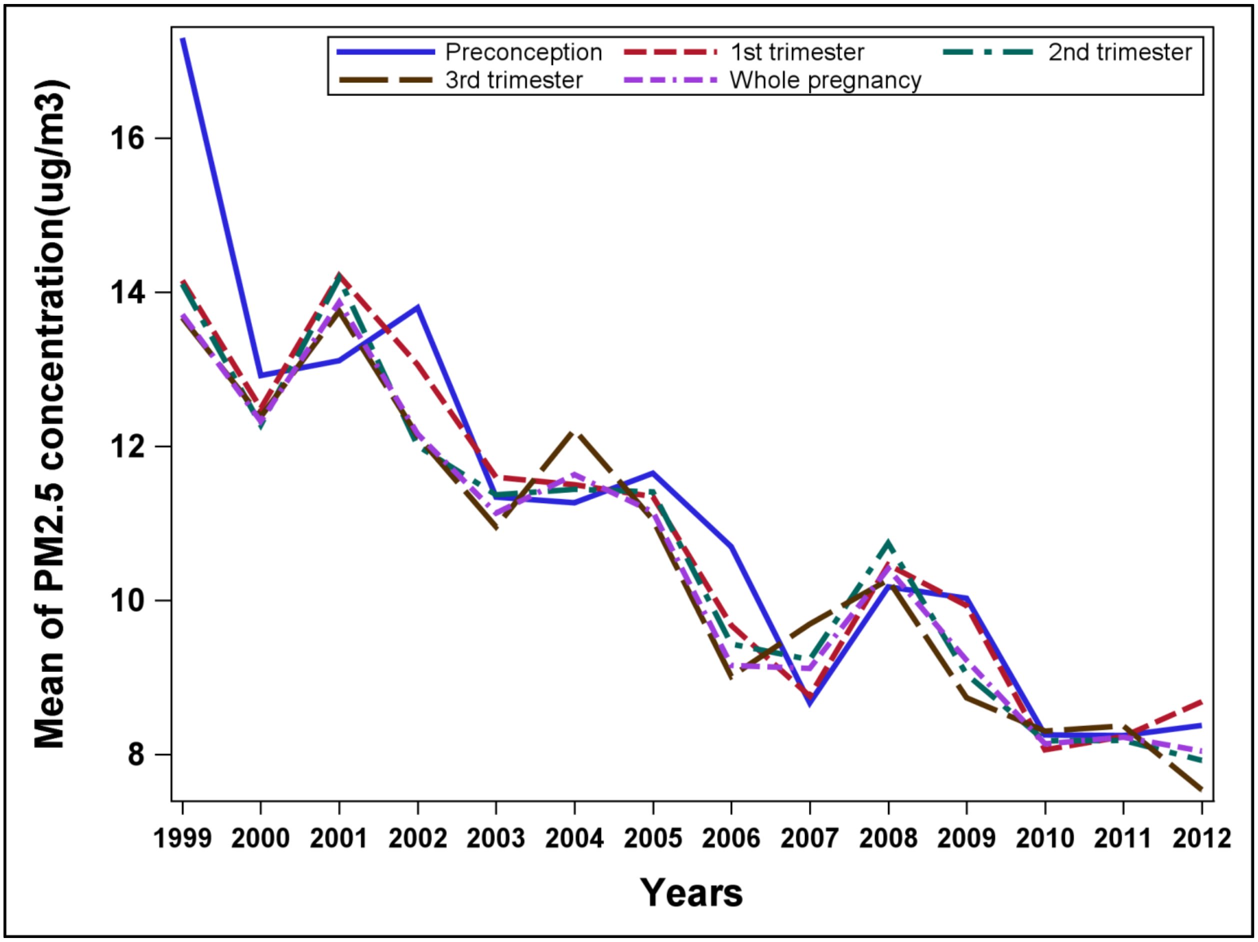

Figure S2. Mean Ambient $PM_{2.5}$ Exposure over Time in a Subset of 5,059 Mothers in the Boston Birth Cohort from 1999-2012
